# A NiCo oxide/NiCo sulfate hollow nanowire-coated separator: a versatile strategy for polysulfide trapping and catalytic conversion in high-performance lithium-sulfur batteries†

**DOI:** 10.1039/d5ra00172b

**Published:** 2025-04-01

**Authors:** Jiarui Liu, Xinhai Wang, Tinghong Gao, Wensheng Yang, Qinyan Jian, Bingxian Li, Lishan He, Yunjun Ruan

**Affiliations:** a Institute of Advanced Optoelectronic Materials and Technology, College of Big Data and Information Engineering, Guizhou University Guiyang 550025 China yjruan@gzu.edu.cn

## Abstract

Lithium-sulfur batteries (LSBs) are highly anticipated due to their remarkable theoretical specific energy and energy density. Nevertheless, the polysulfide shuttle effect severely curtails their cycle life, posing a significant obstacle to commercialization. Herein, we introduce nickel-cobalt oxide/nickel-cobalt sulfate hollow nanowires (NCO/NCSO-HNWs) as a separator modification material. The ingeniously designed hollow nanostructure of NCO/NCSO-HNWs endows it with a profusion of adsorption and catalytic active sites. This unique feature enables it to not only physically adsorb lithium polysulfides (LiPSs) but also catalytically convert them, thereby remarkably enhancing the anchoring and conversion efficiency of LiPSs. The LSBs equipped with NCO/NCSO-HNWs-modified separators exhibit an outstanding initial capacity of 1260 mA h g^−1^ at 0.2C. Even after 100 cycles, a high capacity of 956 mA h g^−1^ is retained, corresponding to an impressive retention rate of 75.9%. Notably, at 1C, after enduring 500 cycles, the discharge capacity still stabilizes at 695 mA h g^−1^. The utilization of such hollow nanowire-based separator modification materials represents a novel and effective strategy for elevating the performance of LSBs, holding substantial promise for surmounting the challenges associated with the shuttle effect and expediting the commercialization journey of LSBs.

## Abstract 2

NCO/NCSO-HNWs were synthesized *via* electrospinning, hydrothermal, and annealing methods as a lithium-sulfur battery separator modifier material, which exhibits remarkable capacity and cycling stability.

## Graphical abstract

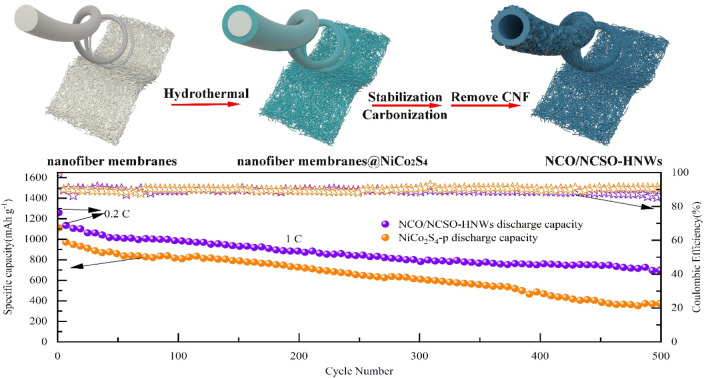

## 1 Introduction

The rapid development of electric vehicles and mobile electronics has spurred the exploration of next-generation high-energy-density secondary batteries to replace the current lithium-ion batteries.^1–3^ However, emerging Na^+^/K^+^/Zn^2+^ secondary batteries and lithium/sodium-sulfur batteries have attracted significant research interest due to their similar metal-ion insertion/extraction storage mechanisms and low-cost raw materials.^4–8^ Among them, lithium-sulfur batteries (LSBs) are one of the most promising candidates because of their high theoretical capacity (1675 mA h g^−1^) and energy density (2600 W h kg^−1^), as well as the abundant sulfur resources in nature, which make them more environmentally friendly.^9–13^ However, as researchers have delved deeper into LSB technology, several challenges to their commercial application have emerged.^14^ These challenges include: (1) the poor electronic and ionic conductivity of sulfur, which limits the high-rate performance of LSBs;^15^ (2) during the charge–discharge processes, sulfur undergoes morphological changes, forming a series of polysulfides. The soluble lithium polysulfides (LiPSs) dissolve into the electrolyte and shuttle between the cathode and anode, resulting in irreversible capacity loss;^16–19^ (3) sulfur undergoes volume expansion during cycling, leading to damage to LSBs.^20,21^

Over the past decade, numerous effective and direct strategies have been explored to limit the diffusion of lithium polysulfides, such as designing sulfur host materials to enhance cycle stability.^22^ Unfortunately, the introduction of host materials reduces the sulfur content in the cathode, thereby lowering the specific capacity and energy density. In contrast, separator modification has been proven to be a cost-effective and efficient approach.^23–27^ Typically, separator modification endows the separator with chemically selective blocking functions, suppressing the migration of soluble LiPSs while accelerating their conversion.^10,28–31^ The blocking performance of the modified layer is closely related to the inherent affinity between the modification material and soluble LiPSs.^32^ Studies have shown that coating carbon materials, such as porous carbon,^33–36^ and graphene oxide,^37^ on separators can enhance the long-term discharge performance of LSBs by inhibiting the polysulfide shuttle effect. Although these carbon-based materials effectively suppress the polysulfide shuttle effect due to their structural characteristics, their weak binding with polysulfides results in poor cycling stability.^38^

Metal compounds with abundant active sites and tunable structures, which exhibit stronger chemical trapping ability for polar LiPSs, have shown significant potential in catalyzing the conversion of LiPSs and effectively inhibiting the shuttle effect.^13^ Among them, nickel-based and cobalt-based composites are promising candidates due to their relatively high conductivity, multiple oxidation states, and diverse crystal structure.^39^ Islam Rakhimbek *et al.* proposed using multifunctional Ni/NiO-embedded carbon nanofibers (Ni/NiO@CNFs) synthesized by electrospinning to enhance the kinetics of LiPS redox reactions and provide extended cyclability by utilizing more efficient active materials.^40^ Therefore, modifying separators with metal compounds is a promising strategy, offering new research perspectives for developing efficient LSBs.^41^

Herein, we have decorated nanofiber membranes with nickel-cobalt sulfides and then removed the CNF by high-temperature treatment to obtain the nickel-cobalt oxides/nickel-cobalt sulfates hollow nanowires (NCO/NCSO-HNWs). The unique hollow structure of NCO/NCSO-HNWs, along with the catalytic conversion of LiPSs by the metal compounds, enables LSBs assembled with the modified separator to exhibit high specific capacity, excellent cycling stability, and outstanding rate performance.

## 2 Experimental section

All chemical reagents were purchased from Shanghai Aladdin Biochemical Technology Co., Ltd and used directly without further purification.

### 2.1 Preparation of NCO/NCSO-HNWs

In brief, a 10% polyacrylonitrile dissolved in *N*,*N*-dimethylformamide (PAN-DMF) was electrospun under 10 kV to form nanofiber membranes. A suitable amount of the nanofiber membranes was cut and used as the substrate for the hydrothermal reaction. 9 mmol of nickel nitrate hexahydrate, 4.5 mmol of cobalt nitrate hexahydrate, 18 mmol of ammonium fluoride, and 18 mmol of thioacetamide were dissolved in 60 mL of deionized water within a 100 mL polytetrafluoroethylene (PTFE) container. After stirring for 30 minutes, the cut nanofiber membrane was fully immersed in the solution. The PTFE container was then sealed and transferred to a stainless-steel autoclave, where it reacted at 150 °C for 7 h. After the mixture was cooled to room temperature, the products were washed several times with deionized water and anhydrous ethanol, then transferred to a vacuum oven at 60 °C and dried overnight. The fully dried nanofiber membranes were placed in a muffle furnace with a heating rate of 1 °C min^−1^ to 250 °C and held for 2 h. After that, the product was transferred to a tube furnace for carbonization under nitrogen protection at 550 °C for 2 h. Finally, the product was subjected to a 2 °C min^−1^ heating rate in the air to 550 °C and held for 3 hours, yielding the NCO/NCSO-HNWs.

### 2.2 Preparation of NiCo_2_S_4_-p

9 mmol of nickel nitrate hexahydrate, 4.5 mmol of cobalt nitrate hexahydrate, 18 mmol of ammonium fluoride, and 18 mmol of thioacetamide were dissolved in 60 mL of deionized water within a 100 mL PTFE cup. After stirring for 30 minutes, the PTFE container was then sealed and transferred to a 100 mL stainless-steel autoclave, where it reacted at 150 °C for 7 h. After the reaction, the NiCo_2_S_4_ powder (NiCo_2_S_4_-p) was obtained by centrifugation, washed three times with ethanol and deionized water, and dried in a vacuum oven at 60 °C for 12 h.

### 2.3 Preparation of separator and cathode material

First, 70% NCO/NCSO-HNWs or NiCo_2_S_4_-p, 20% acetylene black, and 10% polyvinylidene difluoride (PVDF) were added to *N*-methyl-2-pyrrolidone (NMP) to form a slurry under grinding. The slurry was then coated onto a separator and dried for 8 h.

Sulfur powder and acetylene black (S/C) were mixed in a 7 : 3 mass ratio and heated at 155 °C under an Ar atmosphere for 12 h. Then, 70% of S/C, 20% acetylene black, and 10% PVDF were mixed in NMP to form a slurry, which was coated onto aluminum foil. After drying in a vacuum oven at 60 °C for 12 h, the S/C cathode was prepared for further battery assembly. The sulfur content in the cathode is 67%, and the sulfur loading is 1.2 mg cm^−2^.

### 2.4 Battery assembly and testing

The battery was assembled in a CR2032 coin cell, with the cathode shell, the S/C cathode, the NCO/NCSO-HNWs-modified ceramic separator, the lithium foil anode, a spring, gasket, and the anode shell placed in sequence. 15 μL of electrolyte was added to each side of the separator, and the assembly was sealed using a battery packaging machine. All assembly steps were performed in a glove box. The electrolyte was 1 M lithium bis(trifluoromethanesulfonyl)imide (LiTFSI) dissolved in a mixture of 1,3-dioxolane (DOL) and 1,2-dimethoxyethane (DME) (v/v = 1 : 1), with 2 wt% lithium nitrate added.

### 2.5 Characterization techniques

The crystallinity of the samples was studied using X-ray diffraction (XRD) with a PANalytical Empyrean X-ray diffractometer. The elemental valence states and chemical environments were determined using X-ray photoelectron spectroscopy (XPS) with a Thermo Fisher K-Alpha Plus system. The morphology and microstructure of the samples were characterized using field emission scanning electron microscopy (FESEM, HITACHI SU8010) and transmission electron microscopy (TEM, JEOL JEM-2100f). The specific surface area and average pore diameter of the samples were analyzed using a Micromeritics ASAP 2460 automatic surface area and porosity analyzer.

## 3 Results and discussion

**Fig. 1:**
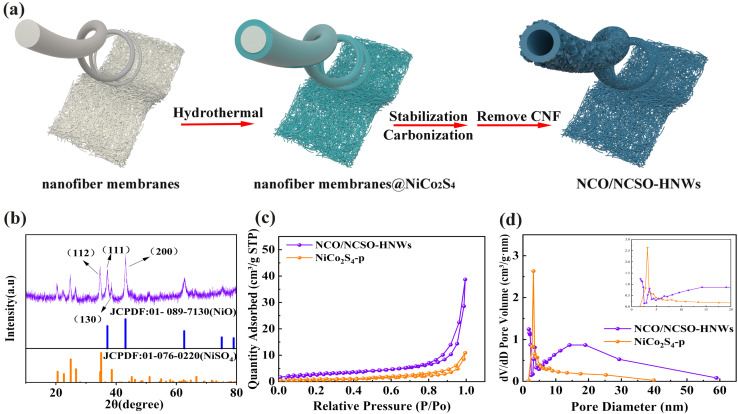
(a) Schematic synthetic processes of NCO/NCSO-HNWs. Structural characterization of CO/NCSO-HNWs: (b) XRD patterns, (c) N_2_ adsorption/desorption isotherms, and (d) pore size distribution (inset is the pore size distribution).

The synthesis process of NCO/NCSO-HNWs is illustrated in Fig. 1a. Nanofiber membranes were first prepared by electrospinning, followed by the growth of a layer of nickel-cobalt sulfide on their surface *via* a hydrothermal method. The nanofiber membranes were then stabilized at 250 °C, followed by carbonization at 550 °C. Finally, the carbon nanofibers were removed by calcination in air at 550 °C, forming nickel-cobalt oxides/nickel-cobalt sulfates hollow nanowires.

The crystal structure of the samples was examined by X-ray diffraction (XRD), as shown in Fig. 1b. The diffraction peaks of NCO/NCSO-HNWs at 37.1°, 43.1°, 62.8°, and 75.3° correspond to the (111), (200), (220), and (311) crystal planes of NiO (JCPDF no. 01-089-7130). The remaining diffraction peaks at 20.3°, 22.1°, 24.8°, 26.6°, 34.7°, 38.5°, and 51.0° align well with the (110), (020), (111), (021), (200), (130), and (222) crystal planes of NiSO_4_ (JCPDF no. 01-076-0220). Due to the similar ionic radii of Ni and Co, they easily form compounds with the same crystal structures. Therefore, the sample is likely a mixture of nickel–cobalt oxides and nickel-cobalt sulfates. Fig. S1 (see ESI†) shows the XRD pattern of NiCo_2_S_4_-p, which matches well with the spinel-structured NiCo_2_S_4_ (JCPDF no. 043-1477).

The specific surface area and pore size distribution of NCO/NCSO-HNWs and NiCo_2_S_4_-p were characterized using N_2_ adsorption–desorption isotherms. As shown in Fig. 1c, both samples exhibit a typical type IV isotherm, with an H3-type hysteresis loop at high relative pressures (*P*/*P*_0_ = 0.9–1.0), indicating the presence of a significant amount of irregular slit-shaped mesopores in the materials. Fig. 1d further analyzes the pore size distribution of the two samples. Notably, the inset in Fig. 1c shows that NCO/NCSO-HNWs and NiCo_2_S_4_-p exhibit strong absorption peaks at 0–2 nm and 10–50 nm, respectively, indicating significant differences in their pore size distributions. As shown in Table 1, the specific surface area of NCO/NCSO-HNWs is 10.8333 m^2^ g^−1^, higher than that of NiCo_2_S_4_-p (3.6588 m^2^ g^−1^), thereby increasing the surface area available for the adsorption of additional electrolyte ions. Moreover, the micropore volume of NCO/NCSO-HNWs is 0.001009 cm^3^ g^−1^, while the mesopore volume is 0.05886 cm^3^ g^−1^. The larger pore volume of NCO/NCSO-HNWs facilitates the reduction of ion diffusion distances and enhances the catalytic conversion of LiPSs. Additionally, the average mesopore size of NCO/NCSO-HNWs is larger than that of NiCo_2_S_4_-p, which is favorable for the diffusion of electrolyte ions and further promotes the improvement of electrochemical performance.

**Table 1:** Physical properties of NCO/NCSO-HNWs and NiCo_2_S_4_-p

Samples	Specific surface area (m^2^ g^−1^)	Pore volume (cm^3^ g^−1^)		Pore size (nm)
		Micropore	Other	
NCO/NCSO-HNWs	10.8333	0.001009	0.05886	25.2435
NiCo_2_S_4_-p	3.6588	0.000180	0.01672	20.1730

**Fig. 2:**
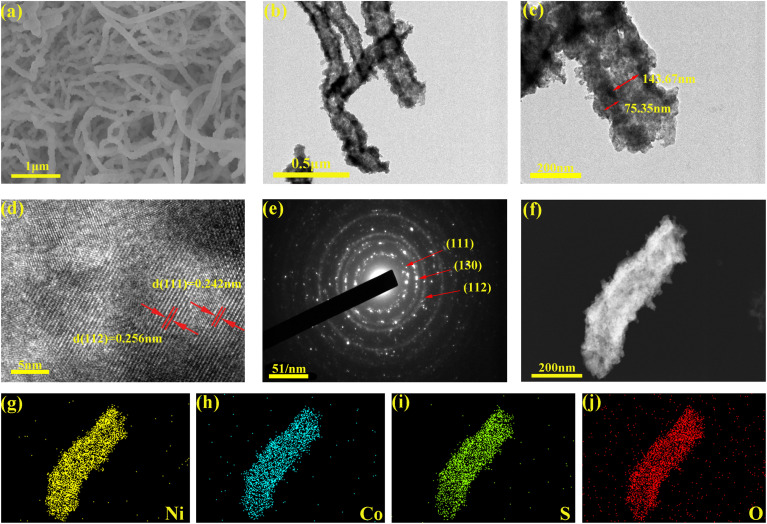
(a) SEM, (b and c) TEM, (d) HRTEM, (e) SAED, and (f–j) elemental mapping images of NCO/NCSO-HNWs.

The microscopic morphology of NCO/NCSO-HNWs was characterized by SEM and TEM. Fig. 2a shows that NCO/NCSO-HNWs form a 3D network structure made up of nanowires, with lengths ranging from several to tens of micrometers. Fig. S2† displays the SEM image of NiCo_2_S_4_ particles. The TEM image in Fig. 2b reveals that these nanowires are hollow and have a rough surface. Fig. 2c demonstrates that the inner diameter of the NCO/NCSO-HNWs tubes is approximately 143.67 nm, with a tube thickness of about 75.35 nm. Fig. 2d, a high-resolution TEM image, displays the lattice fringes of NCO/NCSO-HNWs with the spacing of 0.256 nm and 0.242 nm, which correspond to the (112) crystal plane of nickel sulfate and the (111) crystal plane of nickel oxide, respectively. Fig. 2e presents the selected area electron diffraction (SAED) pattern of NCO/NCSO-HNWs, where several bright rings indicate its polycrystalline nature. Furthermore, Fig. 2f–j show the energy-dispersive X-ray spectroscopy (EDS) element mapping of NCO/NCSO-HNWs, revealing that the Ni, Co, S, and O elements are uniformly distributed throughout the nanowires, with no signs of heterojunctions or local aggregation. Moreover, Fig. S3† shows the optical photo and the cross-section SEM image of the NCO/NCSO-HNWs-coated separator, where the thick of NCO/NCSO-HNWs layer is about 40.35 μm.

**Fig. 3:**
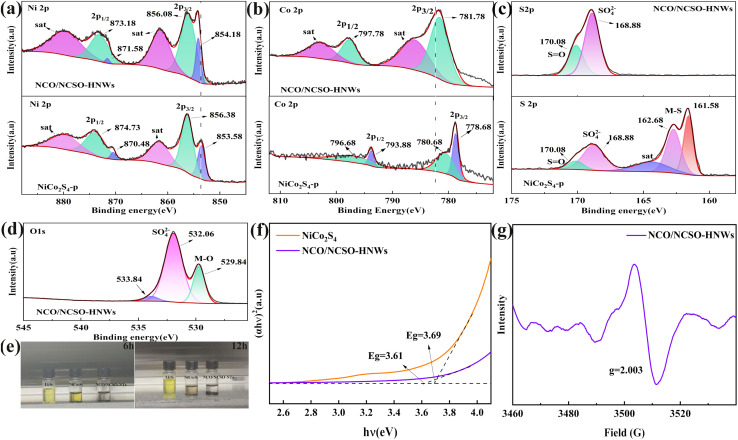
XPS spectra of (a) Ni 2p, (b) Co 2p, (c) S 2p, and (d) O 1s of the NCO/NCSO-HNWs and NiCo_2_S_4_-p. (e) Photographs and (f) bandgap diagrams calculated from UV-vis absorption spectra of the adsorption of blank Li_2_S_6_-DME/DOL solutions containing NCO/NCSO-HNWs and NiCo_2_S_4_-p powder, respectively. (g) The EPR of NCO/NCSO-HNWs.

To further analyze the elemental composition and valence states of NCO/NCSO-HNWs and NiCo_2_S_4_-p, XPS characterization was performed. As shown in Fig. S4,† the measured survey spectra display characteristic peaks for Ni, Co, S, and O elements in NCO/NCSO-HNW composite. In the Ni 2p spectrum (Fig. 3a), NCO/NCSO-HNWs show two spin–orbit doublets at 854.18 and 871.58 eV, which are attributed to Ni 2p_3/2_ and Ni 2p_1/2_ of the Ni–O bond.^42^ Peaks at 856.08 eV and 873.18 eV correspond to the Ni 2p_3/2_ and Ni 2p_1/2_ of NiSO_4_.^43^ The remaining two peaks are attributed to satellite peaks of Ni.^44^ For NiCo_2_S_4_-p, peaks at 853.58 and 870.48 eV correspond to the Ni 2p_3/2_ and Ni 2p_1/2_ of the Ni–S bond.^8^ Peaks at 856.38 and 874.73 eV are attributed to Ni^2+^ in NiSO_4_, arising from the oxidation of the sulfide in air.^43^

In the high-resolution Co 2p spectrum (Fig. 3b), NCO/NCSO-HNWs show two peaks at 781.78 and 797.78 eV, which correspond to Co^2+^ in the Co–O bond, specifically the Co 2p_3/2_ and Co 2p_1/2_. The remaining peaks are satellite peaks resulting from spin–orbit splitting. For NiCo_2_S_4_-p, peaks at 778.68 and 793.88 eV are attributed to Co^3+^ 2p_3/2_ and 2p_1/2_ in cobalt sulfide.^45^

Fig. 3c exhibits two characteristic peaks at 168.88 and 170.08 eV, which are assigned to S–O and S=O bonds, indicating the presence of sulfate in both samples. For NiCo_2_S_4_-p, binding energies of 161.58 and 162.68 eV are assigned to the S 2p_3/2_ and S 2p_1/2_ of divalent metal sulfides.^46^

In Fig. 3d, the O 1s spectrum of NCO/NCSO-HNWs shows three peaks: the peak at 529.84 eV corresponds to the M−O bond, specifically the Ni–O bond; the peak at 532.06 eV corresponds to the sulfate ion (SO_4_^2−^); and the peak at 533.84 eV corresponds to the adsorbed water.^47^

To investigate the adsorption and immobilization of soluble polysulfides on the prepared NCO/NCSO-HNWs and NiCo_2_S_4_-p, Li_2_S_6_ adsorption experiments were performed. Specifically, 30 mg of as-prepared material was added to 5 mL of 0.5 mM Li_2_S_6_ DOL/DME (v/v = 1 : 1) solution. As shown in Fig. 3e, the color of NCO/NCSO-HNWs changed from yellow-brown to transparent after 6 h and 12 h, whereas the NiCo_2_S_4_-p still appeared slightly yellow-brown after 12 h. Thus, NCO/NCSO-HNWs exhibit superior polysulfide conversion and adsorption capabilities.

In Fig. S5,† the ultraviolet-visible (UV-vis) spectra demonstrate that there is a substantial decline in the absorption intensity of the Li_2_S_6_ solutions within the wavelength range of 300 to 500 nm, after the addition of NiCo_2_S_4_-p and NCO/NCSO-HNWs powders for a duration of 12 hours. Notably, the intensity of the absorption peak corresponding to NCO/NCSO-HNWs is lower than that of NiCo_2_S_4_-p, implying that NCO/NCSO-HNWs exhibit superior capacity to anchor and transform polysulfides *via* chemisorption, as compared to NiCo_2_S_4_-p.^48^Fig. 3f provides evidence that the band gap of NCO/NCSO-HNWs is not only lower but also more optimally configured when compared to that of NiCo_2_S_4_-p, strongly suggesting that a battery incorporating NCO/NCSO-HNWs can achieve superior capacity and enhanced electron transport efficiency.

Fig. 3g presents the electron paramagnetic resonance (EPR) spectrum of the NCO/NCSO-HNWs. A distinct peak at 3510 G corresponds to a *g*-factor of 2.003, which is close to the free electron value (2.0023), indicating the presence of delocalized unpaired electrons. This signal likely originates from oxygen vacancies within the nickel-cobalt oxide/sulfate mixed structure. The spectrum exhibits a symmetric single-peak profile with no observable hyperfine splitting. When employed as a separator coating in LSB, these paramagnetic centers effectively anchor lithium polysulfides (LiPS) through Lewis acid–base interactions or chemisorption, thereby suppressing the “shuttle effect” and significantly enhancing cycling stability and capacity retention of the battery system.^49^

Fig. S6† compares the zeta potential distributions of NCO/NCSO-HNWs and NiCo_2_S_4_-p. The higher zeta potential (+7.47 mV) of NCO/NCSO-HNWs enhances colloidal stability and dispersion, preventing nanowire aggregation during slurry coating and enabling uniform separator film formation, which optimizes Li^+^ transport pathways. Furthermore, the positively charged surface of NCO/NCSO-HNWs facilitates electrostatic interactions with negatively charged lithium polysulfides.

**Fig. 4:**
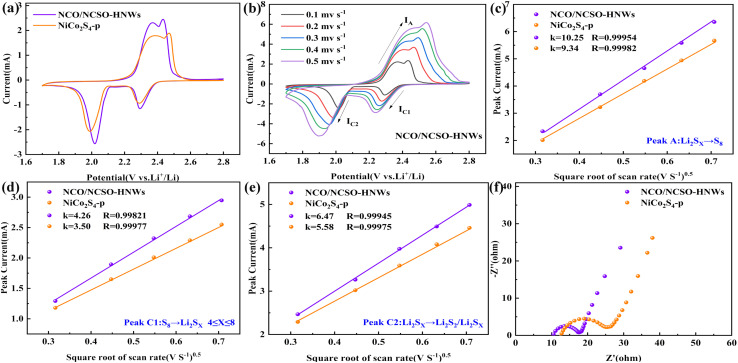
Electrochemical kinetics of NCO/NCSO-HNWs and NiCo_2_S_4_-p: (a) CV curves at 0.1 mV s^−1^, (b) CV curves at different scan rates, (c–e) the corresponding linear relationships between the peak redox currents and the scan rates, and (f) Nyquist plots.

The electrochemical kinetics of LSBs with NCO/NCSO-HNWs and NiCo_2_S_4_-p coated separators were studied through CV (cyclic voltammetry) and EIS (electrochemical impedance spectroscopy) tests. Fig. 4a shows that NCO/NCSO-HNWs have a larger CV area for the oxidation-reduction peaks compared to NiCo_2_S_4_-p, indicating that NCO/NCSO-HNWs enhance the adsorption of LiPSs, improve the utilization of active materials, and effectively hinder the shuttle effect of polysulfides. The oxidation peaks at 2.37 and 2.42 V correspond to the conversion of Li_2_S_2_/Li_2_S to long-chain LiPSs, followed by the conversion to S_8_. In the reduction reaction, the peaks at 2.28 and 2.01 V correspond to the conversion of S_8_ to long-chain LiPSs and further conversion to the final products Li_2_S_2_/Li_2_S.

Fig. 4b displays the CV curves of NCO/NCSO-HNWs at different scan rates. As the scan rate increases, the oxidation peak shifts to higher potentials, while the reduction peak shifts in the opposite direction. According to the classical Randles–Sevcik equation, the lithium-ion diffusion rate can be determined from the following equation:^50^

1 *I*_p_ = 2.69 × 10^5^*n*^1.5^*aD*_Li_^+0.5^*v*^0.5^*C*_Li_

where *I*_p_ is the peak current, *n* is the number of electrons, *a* is the active electrode area, *D*^+^_Li_ is the lithium-ion diffusion coefficient, *C*_Li_ is the lithium-ion concentration in the cathode, and *ν* is the scan rate. The relationship between *I*_p_ and *v*^0.5^ yields a fitted line with a slope (Fig. 4c–e). NCO/NCSO-HNWs exhibit a larger slope, indicating a faster Li^+^ diffusion rate. This may be due to the larger specific surface area and more pore structures in NCO/NCSO-HNWs, which facilitate the diffusion of the electrolyte ions. Additionally, the nickel-cobalt compounds continuously catalyze the transformation of polysulfides, alleviating the shuttle effect. By fitting the cyclic voltammetry (CV) curves at different scan rates, the capacitive and diffusive contributions in the NCO/NCSO-HNWs and NiCo_2_S_4_-p batteries were obtained, as shown in Fig. S7.† At each scan rate, the diffusive contribution of the NCO/NCSO-HNWs separator is higher than that of the NiCo_2_S_4_-p separator. Even at a scan rate of 0.5 mV s^−1^, the diffusive contribution of the NCO/NCSO-HNWs separator remains at a relatively high level of 72.5%. A higher diffusive contribution indicates that the diffusion behavior dominates the lithium storage reaction. The separator modified with NCO/NCSO-HNWs improves the diffusion kinetics, which is the reason for the high capacity and excellent rate performance of the battery with the NCO/NCSO-HNW-modified separator.^51^

Fig. 4f exhibits the Nyquist plots, where NCO/NCSO-HNWs exhibit lower internal resistance and charge transfer resistance compared to NiCo_2_S_4_-p. Fig. S8 and Table S1† show the results of the equivalent circuit fitting based on the EIS curves. The charge transfer resistance of the battery using the NCO/NCSO-HNWs separator is 8.75 Ω, which is lower than that of the battery using the Co–NiCo_2_S_4_-p separator (9.98 Ω). This indicates that modifying the separator with the NCO/NCSO-HNWs composite can reduce the interfacial charge transfer resistance.^52^ Additionally, the excellent catalytic transformation ability of the nickel-cobalt compounds towards polysulfides leads to a lower charge transfer resistance. Therefore, the NCO/NCSO-HNWs coated separator enhances the adsorption of polysulfides and accelerates their catalytic transformation.

**Fig. 5:**
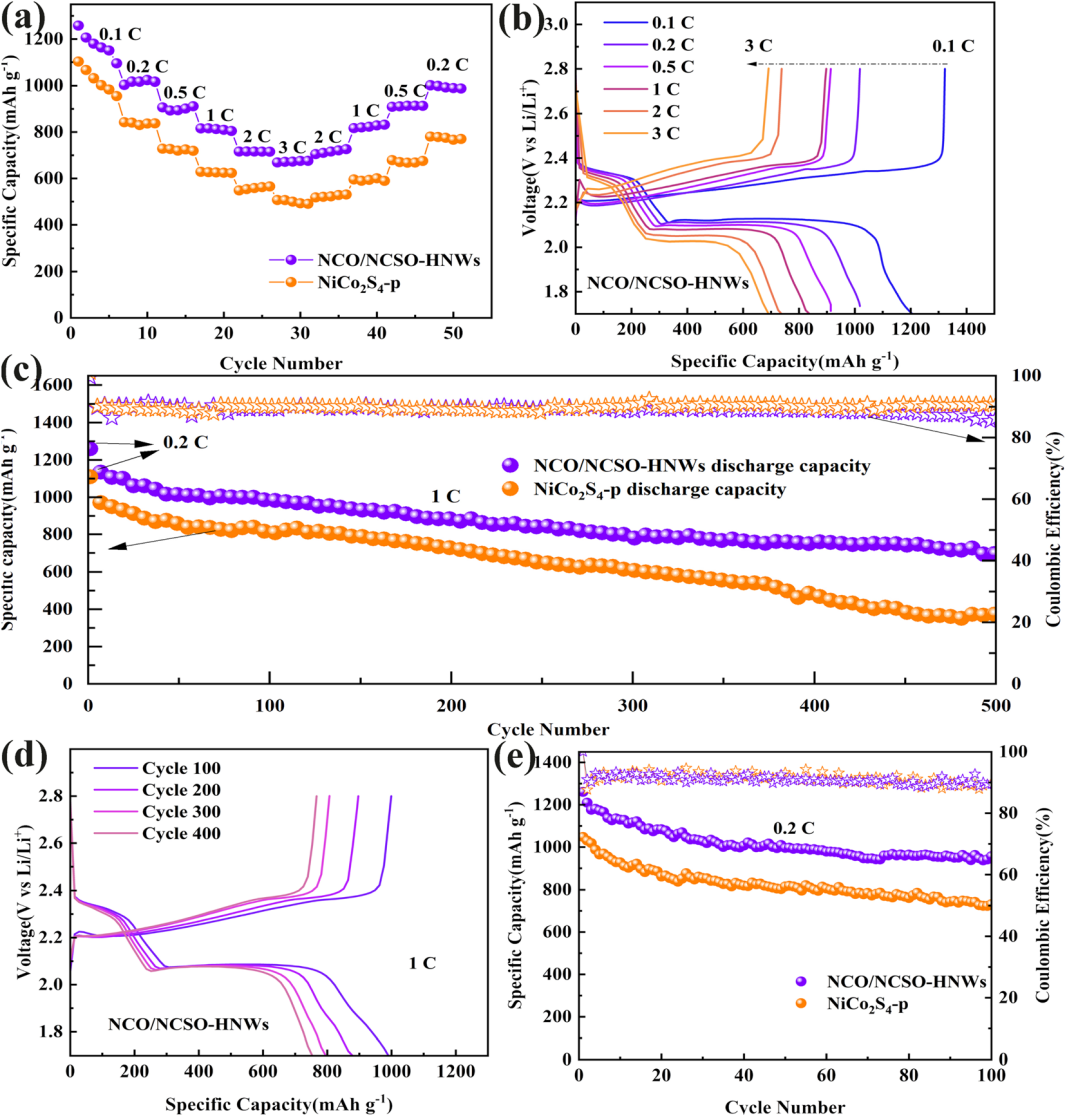
Electrochemical stability of NCO/NCSO-HNWs and NiCo_2_S_4_-p. (a) Capacity *versus* current density, (b) GCD curves of NCO/NCSO-HNWs at various current densities, (c) 500 cycles at 1C, (d) GCD curves at 1C in various cycles of NCO/NCSO-HNWs and (e) 100 cycles at 0.2C.

Fig. 5a reveals that the specific discharge capacities of NCO/NCSO-HNWs at different current densities of 0.1, 0.2, 0.5, 1, 2, and 3C are 1257, 1023, 901, 816, 717, and 672 mA h g^−1^, respectively. When the current density returns to 0.2C, the discharge capacity is maintained at 987 mA h g^−1^. In comparison, NiCo_2_S_4_-p exhibits specific discharge capacities of 1102, 842, 727, 626, 562, 499, and 775 mA h g^−1^ at the same current densities. This indicates that NCO/NCSO-HNWs have better rate performance and retain a higher specific capacity at higher current densities.

Fig. 5b presents the galvanostatic charge–discharge (GCD) curves of NCO/NCSO-HNWs at different current densities, showing that the oxidation/reduction peak potentials shift slightly with the increase in current density. At a high current density of 1C, Fig. 5c shows that NCO/NCSO-HNWs achieve an initial capacity of 1131 mA h g^−1^. After 500 cycles, NCO/NCSO-HNWs still retain a capacity of 695 mA h g^−1^, corresponding to a capacity retention of 60%. In contrast, NiCo_2_S_4_-p shows an initial capacity of 991 mA h g^−1^, but after 500 cycles, the specific discharge capacity drops to only 373 mA h g^−1^, resulting in a much lower capacity retention of 38%.

Fig. 5d displays the GCD curves of NCO/NCSO-HNWs at the 100th, 200th, 300th, and 400th cycles at 1C, showing that NCO/NCSO-HNWs maintain a relatively stable charge–discharge platform even after many cycles and indicating its excellent cycle stability. In comparison, Fig. S9† shows the galvanostatic charge–discharge (GCD) curves of NiCo_2_S_4_-p at a current density of 1C. Fig. 5e compares the cycling stability of NCO/NCSO-HNWs and NiCo_2_S_4_-p at a low current density of 0.2C. NCO/NCSO-HNWs show a higher initial capacity of 1260 mA h g^−1^ and retain a high capacity of 956 mA h g^−1^ after 100 cycles. In contrast, NiCo_2_S_4_-p drops from an initial capacity of 1047 to 731 mA h g^−1^. Fig. S10† displays morphological and structural characterization results of the C/S cathode before and after cycling. Table S2† shows the electrochemical performance of NCO/NCSO-HNWs compared with other recently published lithium-sulfur batteries.

These results demonstrate that NCO/NCSO-HNWs can effectively suppress the shuttle effect of LiPSs and reduce capacity loss, thus improving cycling stability at both high and low current densities. The superior performance of NCO/NCSO-HNWs is attributed to its unique structure, which facilitates better polysulfide adsorption and catalytic conversion.

## 4. Conclusion

In summary, we have successfully fabricated nickel-cobalt oxide/nickel-cobalt sulfate hollow nanowires (NCO/NCSO-HNWs) through a meticulously designed two-step process. Firstly, nickel-cobalt sulfide-coated carbon nanofibers are synthesized *via* hydrothermal treatment.

Subsequently, the carbon fibers are removed by high-temperature annealing, giving rise to the NCO/NCSO-HNWs. When applied as a coating on the separator of lithium-sulfur batteries, NCO/NCSO-HNWs demonstrate remarkable efficacy. The hollow nanowire structure functions as a reservoir of physical adsorption sites, effectively confining the shuttle effect of LiPSs. Simultaneously, the nickel and cobalt compounds within the nanowires furnish copious chemical active sites. These sites not only impede the migration of LiPSs but also actively promote their catalytic transformation into less soluble species. Consequently, the lithium-sulfur batteries assembled with NCO/NCSO-HNWs-modified separators exhibit an initial specific capacity of 1260 mA h g^−1^ at 0.2C. Even after 300 cycles at 1C, a remarkable capacity retention of approximately 70% is achieved. After a more extensive 500 cycles at 1C, the battery still delivers a stable capacity of 695 mA h g^−1^. This research pioneers a reliable and reproducible methodology for the fabrication of nickel-cobalt composite nanotube-based separators, offering a viable and promising avenue for the development of high-performance and long-lasting lithium-sulfur batteries.

## Data availability

The datasets generated and analyzed in this current study are available from the corresponding author upon reasonable request.

## Author contributions

Jiarui Liu: writing – original draft, review & editing, visualization, formal analysis, data curation. Xinhai Wang: investigation, methodology, resources. Tinghong Gao: funding acquisition, resources, supervision. Wensheng Yang: funding acquisition, resources, supervision. Qinyan Jian: visualization, validation, investigation. Bingxian Li: visualization, validation, investigation. Lishan He: visualization, validation, investigation. Yunjun Ruan: funding acquisition, resources, supervision, project administration.

## Conflicts of interest

No conflict of interest exists in the submission of this manuscript, and the manuscript is approved by all authors for publication. I would like to declare on behalf of my co-authors that the work described was original research that has not been published previously and is not under consideration for publication elsewhere, in whole or in part. All the authors listed have approved the manuscript that is enclosed.

## Supplementary Material

RA-015-D5RA00172B-s001
