# Supplementary material for: A NiCo oxide/NiCo sulfate hollow nanowire-coated separator: a versatile strategy for polysulfide trapping and catalytic conversion in high-performance lithium-sulfur batteries
Source: RSC Adv. 2025 Apr 1;15(13):9875–83. doi: 10.1039/d5ra00172b (PMC11959455; doi:10.1039/d5ra00172b)
Supplement: RA-015-D5RA00172B-s001 [file RA-015-D5RA00172B-s001.pdf]

Supplementary Information (SI) for RSC Advances.
This journal is © The Royal Society of Chemistry 2025

Supporting Information

NiCo Oxide/NiCo Sulfate Hollow Nanowires-Coated Separator: A Versatile Strategy for Polysulfide Trapping and Catalytic Conversion in High-Performance Lithium-Sulfur Batteries

*Jiarui Liu, Xinhai Wang, Tinghong Gao, Wensheng Yang, Qinyan Jian, Bingxian Li, Lishan He, Yunjun Ruan**

Institute of Advanced Optoelectronic Materials and Technology, College of Big Data and Information Engineering, Guizhou University, Guiyang 550025, China.

**Corresponding author. E-mail: yjruan@gzu.edu.cn (Yunjun Ruan)*

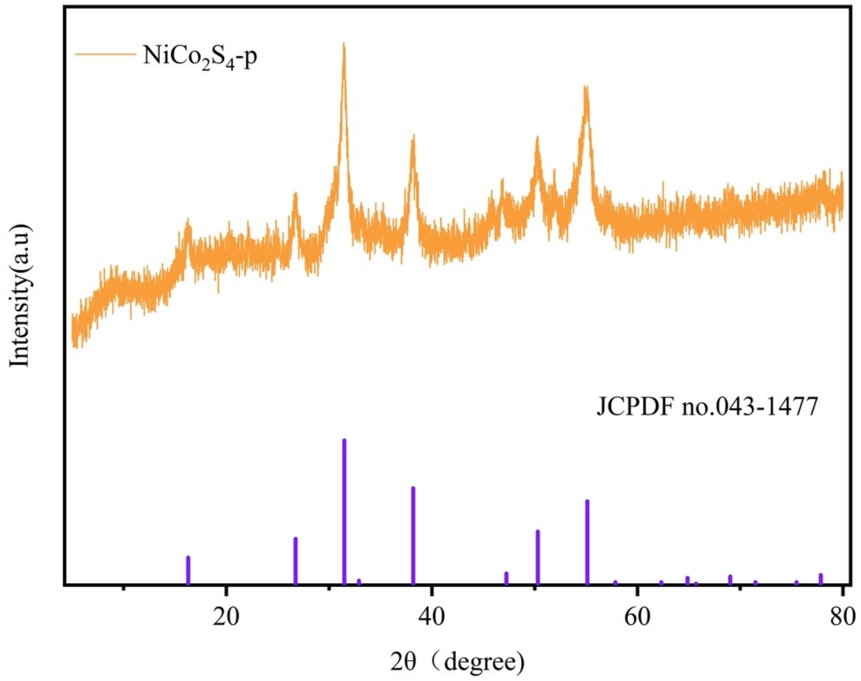

Figure S1. XRD patterns of $NiCo_2S_4$-p.

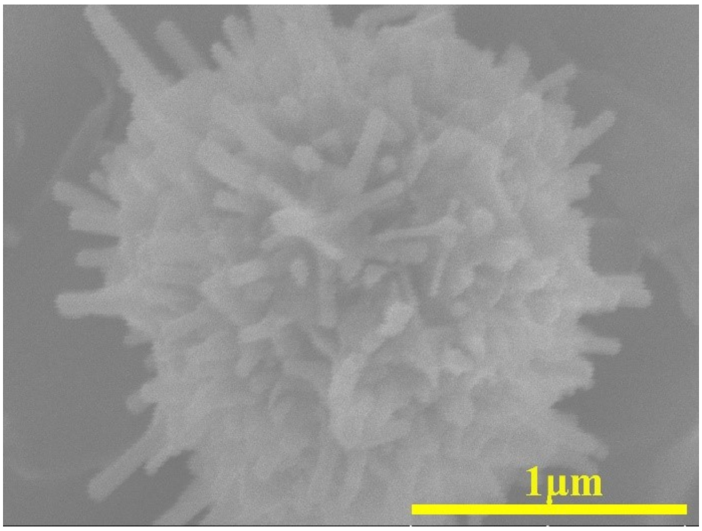

Figure S2. The SEM image of $NiCo_2S_4$-p.

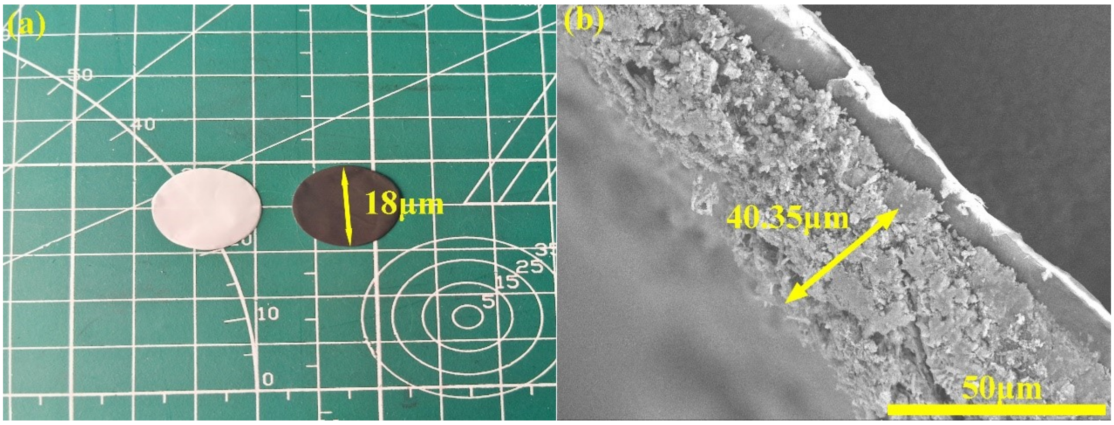

Figure S3. The (a) Optical photo and (b) cross-section SEM image of the NCO/NCSO-HNWs-coated separator.

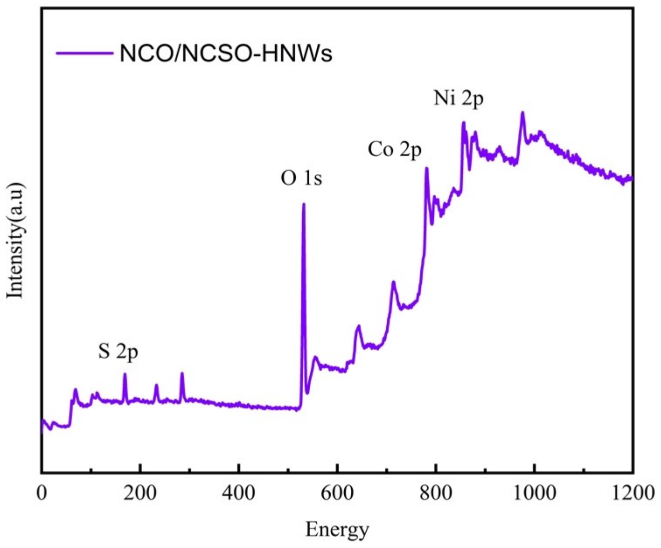

Figure S4. The XPS survey spectra of NCO/NCSO-HNWs.

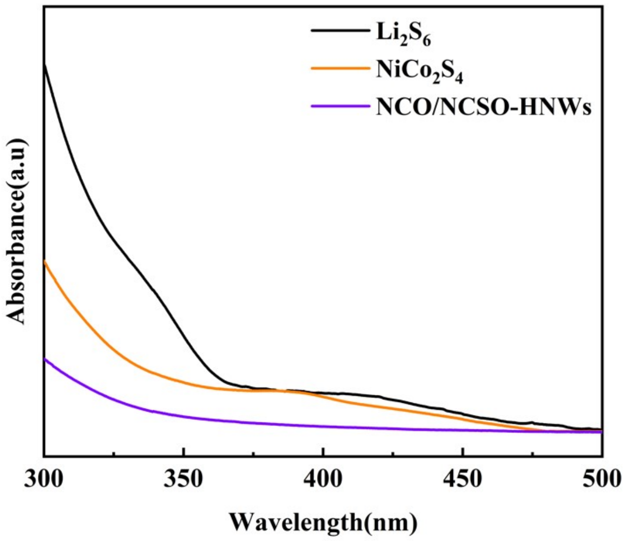

Figure S5. UV−vis absorption spectra of the Li_2S_6-DME/DOL solutions containing $NiCo_2S_4$-p or NCO/NCSO-HNWs powder.

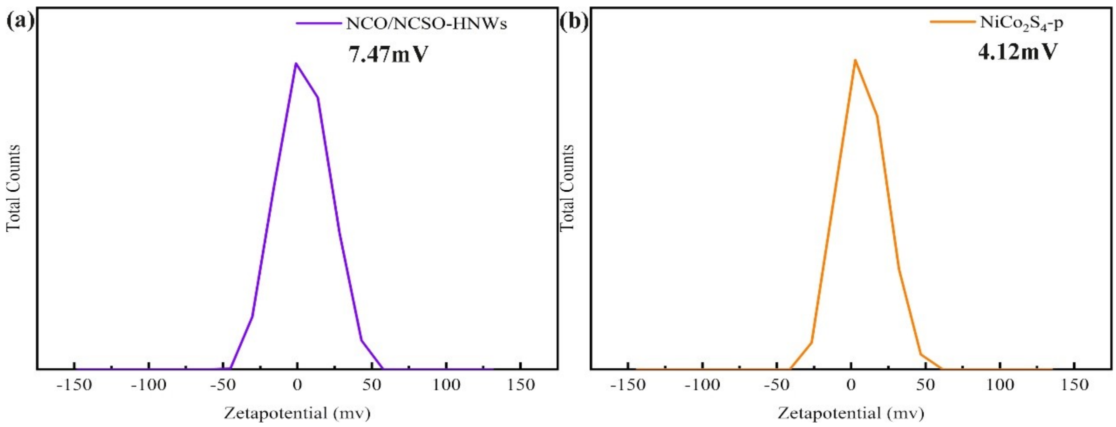

Figure S6. The zeta potential of (a) NCO/NCSO-HNWs and (b) $NiCo_2S_4$-p.

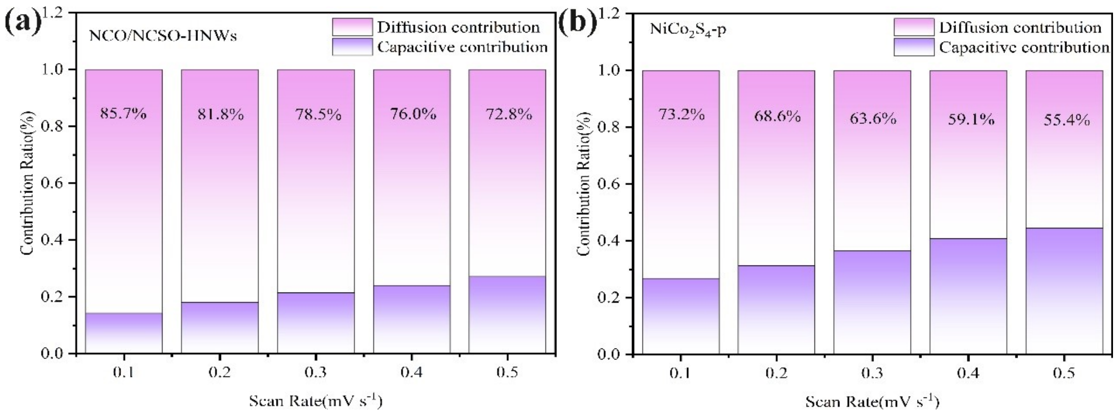

Figure S7. The capacitance contributions and diffusion contributions of (a) NCO/NCSO-HNWs and (b) $NiCo_2S_4$-p.

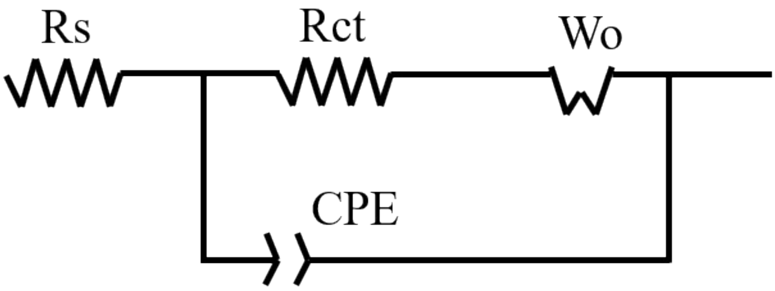

Figure S8. Equivalent circuit diagram.

Table S1. Comparison of equivalent circuit parameters for NCO/NCSO-HNWs and $NiCo_2S_4$-p symmetrical cell

Species	R_s (Ω)	R_{ct} (Ω)
NCO/NCSO-HNWs	13.73	8.75
$NiCo_2S_4$-p	17.93	9.98

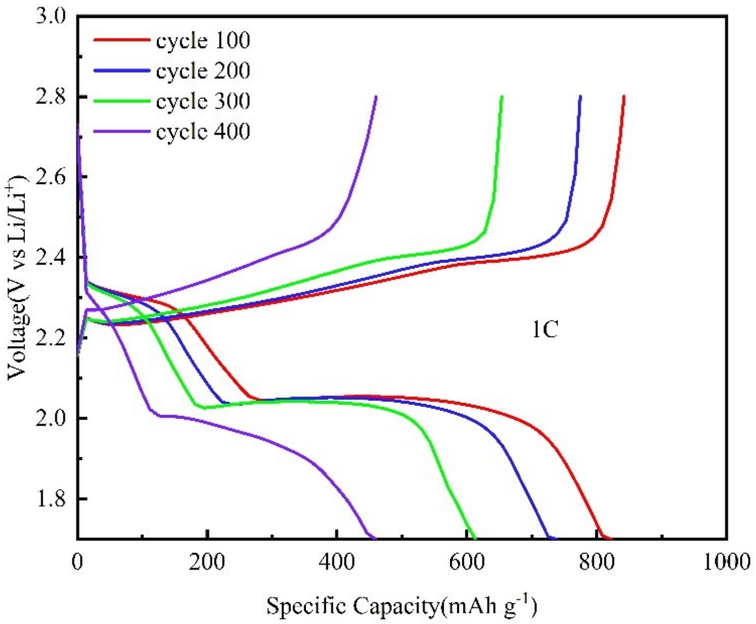

Figure S9. GCD curves at 1 C in various cycles of $NiCo_2S_4$-p.

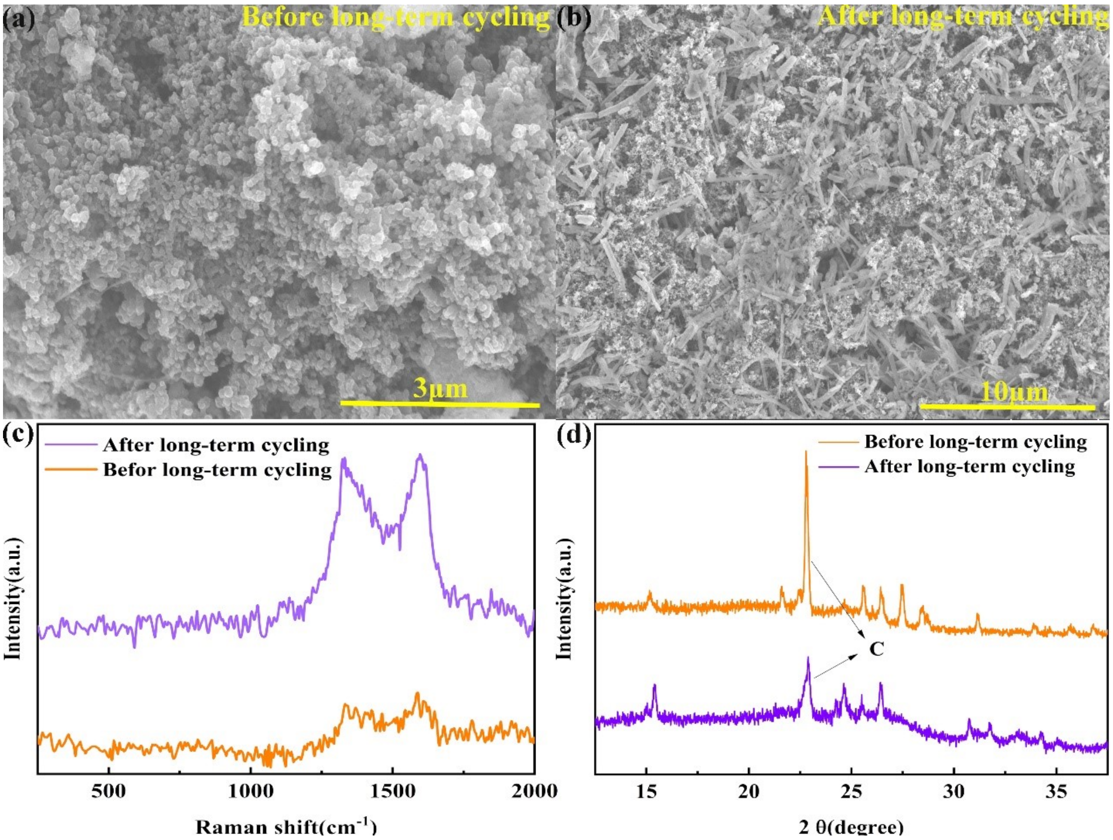

Figure S10. (a,b) SEM, (c) Raman and (d) XRD images of the C/S cathodes before and after long-term cycling.

Figures S10a and **S10b** are the comparative SEM images of the C/S electrode before and after long-term cycling. Evidently, after long-term cycling, a layer of nanorods adheres to the surface of the electrode. These nanorods consist of electrolyte decomposition products and polysulfide reduction products.

Figure **S10c** shows the Raman spectra of the C/S electrode before and after long-term cycling. The ID/IG value after long-term cycling is 1.01, which is higher than the 0.94 value before cycling. This is because during long-term cycling, the insertion and extraction of lithium damage the structure of the carbon material and increase its lattice distortion.

Figure S10d shows the XRD patterns of the C/S electrode before and after long-term cycling. It can be seen that after long-term cycling, the intensity of the diffraction peak of C decreases. This is because the insertion and extraction of lithium disrupt the structure of the carbon material, resulting in a deterioration of the orderliness of the crystal structure.

Table S2 Electrochemical performance of Li-S batteries based on different materials.

Electrode material	Sulfur content (wt %)	Sulfur loading (mg cm^{-2})	Initial capacity (mAh g^{-1})	Cycle number	Capacity after cycling (mAh g^{-1})	Ref.
NGC/PP	70	-	788	200	637 (1 C)	1
PCN@CNT	70	1.5	799	500	545(1C)	2
CFs@PP	60	1.0	1063	500	675(0.5C)	3
Ce-MOF	76	3.5	891.2	300	627(1C)	4
$CoSe_2$@C-N/CNT	65.9	1-1.4	1120	300	761(1C)	5
PP/PMMA	70	0.55	1100	100	613(0.1C)	6
TiN	78	-	1052	250	605(0.5C)	7
VN@NC	75	-	1052	400	694(1C)	8
NCO/NCSO-HNWs	67	1.2	1131	500	695(1C)	This work

References

1. L. He, D. Yang, H. Zhao, L. Wei, D. Wang, Y. Wang, G. Chen and Y. Wei, *Chem. Eng. J.*, 2022, **440**, 135820.
2. X. Hu, T. Huang, S. Wang, S. Lin, Z. Feng, L.-H. Chung and J. He, *Electrochimica Acta*, 2021, **398**, 139317.
3. B. Zheng, L. Yu, Y. Zhao and J. Xi, *Electrochimica Acta*, 2019, **295**, 910–917.
4. Y. Su, W. Wang, W. Wang, A. Wang, Y. Huang and Y. Guan, *J. Electrochem. Soc.*, 2022, **169**, 030528.
5. Y. Luo, H. Bai, B. Li, X. Song, J. Zhao, Y. Xiao, S. Lei and B. Cheng, *J. Alloys Compd.*, 2021, **879**, 160368.
6. C. Deng, Z. Wang, S. Wang, J. Yu, D. J. Martin, A. K. Nanjundan and Y. Yamauchi, *ACS Appl. Mater. Interfaces*, 2019, **11**, 541–549.
7. X. He, Y. Shuai, L. Na, K. Chen, Y. Zhang, Z. Zhang and F. Gan, *Mater. Lett.*, 2018, **215**, 91–94.
8. S. Liu, Y. Liu, X. Zhang, M. Shen, X. Liu, X. Gao, L. Hou and C. Yuan, *Nanomaterials*, 2024, **14**, 656.
